# First-Principles Study on the Adsorption Mechanism of Oxygen on UN(100), (110), and (111) Surfaces

**DOI:** 10.3390/ma19173708

**Published:** 2026-08-31

**Authors:** Tianyu Zhang, Min Zhu, Huang Huang, Longfei Pu, Chengxuan Peng, Longxian Li, Zijian Wang, Boxuan Li

**Affiliations:** College of Nuclear Science and Technology, Naval University of Engineering, Wuhan 430030, China; m25182709@nue.edu.cn (T.Z.); x23182704@nue.edu.cn (L.P.); 33852019007@nue.edu.cn (C.P.); d23182701@nue.edu.cn (L.L.); x24182701@nue.edu.cn (Z.W.); x25182712@nue.edu.cn (B.L.)

**Keywords:** uranium nitride, first principles, surface adsorption, oxygen behavior

## Abstract

Uranium nitride (UN) is considered a promising candidate material for advanced nuclear fuels in Generation IV reactors due to its high thermal conductivity and excellent fission product retention capability. However, the surface corrosion behavior of UN in oxygen-containing environments severely limits its practical application. In this work, first-principles calculations based on density functional theory (DFT) were employed to systematically investigate the adsorption and dissociation behaviors of O_2_ molecules and O atoms on the UN(100), (110), and (111) surfaces. The electronic structure mechanisms underlying the adsorption were elucidated through analysis of surface work function, projected density of states (PDOS), Bader charge, and charge density difference. The calculated results show that O_2_ molecules undergo thermodynamically highly favorable dissociative chemisorption on all three UN surfaces at 0 K, with the O–O bond length stretched to 1.44–1.50 Å, characteristic of a peroxo-like (O_2_^2−^) species, which is intermediate between the superoxide (O_2_^−^, ~1.33 Å) and complete dissociation. The most stable adsorption configurations on each surface are: (100)-H site (−4.05 eV), (110)-B(2) site (−4.81 eV), and (111)-H site (−5.03 eV), with the adsorption strength governed by the surface coordination environment. The adsorption energies of O atoms (−4.5 to −8.2 eV) are significantly higher than those of O_2_ molecules. The order of the most stable O atom adsorption sites is (110)-B(2) site (−8.22 eV) > (100)-H site (−6.97 eV) > (111)-T(U) site (−5.09 eV), which differs from that of O_2_, revealing the efficient O atom trapping effect of the groove structure on the (110) surface. Electronic structure analysis indicates that upon O atom adsorption, the work functions of the (100) and (110) surfaces increase by 0.62 eV and 0.39 eV, respectively, while that of the (111) surface decreases by 0.57 eV, suggesting that the (111) surface is more susceptible to further oxidation. In terms of bonding mechanisms, the interaction between O and U is primarily dominated by p–d hybridization between O-2p and U-6d orbitals. The U-5f orbital participates indirectly through f–d coupling with U-6d, while direct p–f hybridization is enhanced in the case of isolated O atom adsorption. This study provides an atomic-scale theoretical basis for understanding the initial oxidation mechanism of UN surfaces and offers important guidance for the surface protection design of UN-based nuclear fuels.

## 1. Introduction

Since the Fukushima nuclear accident in 2011, research on accident-tolerant fuels (ATFs) aimed at improving the safety characteristics of nuclear fuels has become an increasingly important topic. Recently, several ATFs, such as UN and uranium silicide, have been considered as potential alternatives to uranium oxide (UO_2_), and are promising materials for Generation IV fast neutron reactors [1]. Compared with traditional oxide fuels, UN possesses higher thermal conductivity, melting point, and density, making it well-suited for future high-temperature reactors [2]. Existing studies have mainly focused on the synthesis, performance characterization, environmental qualification, and electronic structure of UN. When exposed to air, UN rapidly reacts with oxygen, forming a complex oxide layer. As the degree of oxidation deepens, the thickness of the oxide layer gradually increases, seriously affecting the service performance of UN [3,4,5]. Improving the anti-oxidation corrosion performance is of great significance for the application of UN. Investigating the oxidation behavior of UN surfaces at the microscopic level can provide theoretical support for the corrosion protection of UN. Modern computer simulation techniques can provide more atomic-level details of UN oxidation, among which density functional theory (DFT), which describes the interactions between atoms, plays a crucial role by achieving a good balance between accuracy and computational cost [6].

In order to investigate the interactions between gaseous O_2_, H_2_O and UN, a series of DFT-based theoretical calculations have been performed. Surface information of UN has been obtained, and some understanding of H and O atom diffusion has been achieved, providing valuable insights into the initial oxidation stage of UN [7,8,9,10,11]. Methodologically, Claisse et al. [12] systematically compared the U-ramping and occupation-matrix control schemes of GGA + U for the strongly correlated U 5f electrons in UN, establishing the DFT + U methodology adopted in the present work. Existing studies indicate that UN reacts actively with oxygen, and O_2_ molecules can dissociate spontaneously on the UN surface [13]. Furthermore, the diffusion of O atoms on the surface and their penetration into subsurface layers and grain boundaries are energetically highly favorable, indicating that UN is readily oxidized when exposed to the environment [14,15]. The energetically favorable occupation of N vacancies by O atoms also indicates that the UN surface is prone to oxidation, and O readily diffuses into the UN crystal structure. The works of Bo demonstrated the possible reactions of H_2_O on the UN surface [9,10], revealing the surface reactions of UN as a potential nuclear fuel if used in nuclear power reactors employing heavy water as a coolant.

Previous studies have shown the adsorption of H_2_O, O_2_, and N_2_ molecules on the surface, revealing the most stable adsorption configurations on the UN(001)surface [16,17]. For H_2_O, its O atom interacts directly with one of the U atoms, while the remaining two H atoms point toward the surface N atoms. The dissociation of O_2_ is thermodynamically highly favorable upon interaction with the UN surface, with each O atom adsorbing at the bridge site between two U atoms. The adsorption energy of O_2_ on the clean UN surface with respect to gaseous O_2_ is negative, ΔE = −8.11 eV, indicating that the interaction between UN and O_2_ is highly reactive. A N_2_ molecule interacts with the UN(001) surface, with one N atom located at the bridge site between two U atoms and the other N atom bonded to a single U atom. The binding energy of N_2_ is ΔE = −0.96 eV, and the distance between the two N atoms increases slightly from 1.11 Å (gas phase) to 1.21 Å (adsorbed phase). A single N atom preferentially adsorbs at the bridge site, with ΔE = −0.68 eV. Similarly, the approach used to study N_2_ on the UN(100) surface can also be applied to the investigation of O_2_ on different UN surfaces.

Beyond the UN(001) surface, recent first-principles work has broadened the theoretical understanding of actinide-fuel oxidation. Chen and Kaltsoyannis [18] employed hybrid-functional and embedded-cluster methods to investigate the {110} surfaces of uranium and mixed actinide dioxides, demonstrating how the local An–O bonding environment governs surface stability. Similarly, Talla Noutack et al. [19] used DFT + U to resolve the structural, electronic, and energetic properties of uranium–americium mixed oxides. On the technological side, Watkins et al. [20] reviewed alloyed and composite fuel architectures for mitigating high-density UN oxidation, underscoring the critical role of surface chemistry in environmental degradation. These internationally representative studies, together with the pioneering UN(001) adsorption work of Zhukovskii et al. [7,15] and Bocharov et al. [8], provide the foundation for the present work.

To date, most DFT studies have been restricted to the (001) surface [7,8,9,10,16,17,18,19,20], and a systematic, facet-resolved comparison of oxygen adsorption—including adsorption energetics, site preference, charge transfer, work function variation, and electronic structure—across the three principal low-index surfaces of UN is still lacking. The present work fills this gap and makes the following contributions: (i) we determine and compare the most stable adsorption configurations and energies of both O_2_ molecules and O atoms on the UN(100), (110), and (111) surfaces, revealing a facet-dependent adsorption anisotropy; (ii) we identify the groove structure of the (110) surface as an efficient O-atom trapping motif, which has not been reported previously; and (iii) we elucidate the bonding mechanism between oxygen and the UN surfaces through a combined analysis of PDOS, Bader charge, charge density difference, and work function.

## 2. Methods and Computational Details

The calculations in this chapter were performed using the VASP [21,22] code based on density functional theory. The interaction between electrons and ions is described using the Projector Augmented Wave (PAW) method. The exchange-correlation energy is calculated using the Perdew–Burke–Ernzerhof (PBE) exchange-correlation functional within the Generalized Gradient Approximation (GGA) [23,24]. The outer electron configuration of uranium is [Rn]5f^3^6d^1^7s^2^, the valence electrons of nitrogen are 2s^2^2p^3^, and those of oxygen are 2s^2^2p^4^. The band structure of UN is dominated by the hybridization between the U 5f/6d orbitals and the N 2p orbital. Due to the strong correlation effects of the U 5f electrons, a Hubbard correction term is required, and the DFT + U method is employed to address this. The difference between the Coulomb energy parameter U and the exchange energy parameter J, U_eff_ = U − J, is significant for the total energy functional. Based on previous studies [11,25], for the UN system, U_eff_ is set to 4 eV (U = 4.5, J = 0.5). This value was chosen because it is the most widely adopted parameterization in previous DFT + U studies of UN surface chemistry, including the pioneering oxygen-adsorption work of Zhukovskii et al. [26] and Bocharov et al. [8], and it has been shown to reproduce the experimental lattice parameter and magnetic properties of UN while ensuring direct comparability with the existing literature [6,25]. The plane-wave cutoff energy is set to 520 eV, which is sufficient to yield well-converged total energies and is consistent with the values used in previous DFT studies of UN surfaces [6,26,27]. During structural optimization, the energy convergence criterion is set to 1 × 10^−4^ eV, and the force convergence criterion is set to 0.02 eV/Å. Slab models of ten atomic layers (five U–N bilayers) were employed for all surface orientations. This choice is comparable to, or slightly thicker than, those used in previous DFT studies of uranium nitride surfaces [11,26,27,28]. While thinner slabs are sufficient for the (100) and (110) surfaces, the (111) surface has a smaller interlayer spacing along the surface normal, necessitating additional layers to converge to a bulk-like interior. A uniform thickness of ten layers was therefore adopted to ensure consistent treatment across all facets, enabling direct comparison of the computed properties. Each surface slab employed a p(1 × 1) lateral unit cell, with in-plane dimensions of 4.893 Å × 4.893 Å for (100), 4.893 Å × 6.919 Å for (110), and 6.919 Å × 6.919 Å for (111). A single adsorbate (one O_2_ molecule, or one O atom) was placed in each supercell, corresponding to a coverage of 0.5 monolayer relative to the surface U sites. At this coverage, the lateral separation between adsorbates in neighboring cells is at least ~4.9 Å, so lateral adsorbate–adsorbate interactions are negligible and the calculations describe the isolated-adsorbate limit. To avoid interactions between adjacent slabs, a vacuum layer of 15 Å is applied. The bottom three atomic layers of the slab model are fixed during structural optimization. In studying the adsorption of oxygen molecules, to eliminate the influence of the initial position of the O_2_ molecule on the relaxation process and to reflect the true geometric configurational changes in the O_2_ molecule during relaxation, the vertical distance between the O_2_ molecule and the nearest surface atom should be greater than the U–O bond length. However, if the oxygen molecule is placed too far from the surface, it may lead to a waste of computational resources. Therefore, the distance between the O_2_ molecule and the UN surface is set to 2 Å prior to relaxation. In all O_2_ adsorption calculations, the molecule was initially placed parallel (flat-lying) to the surface, with its molecular axis aligned along the high-symmetry direction connecting the two surface U atoms of the target adsorption site. The parallel orientation was adopted because it maximizes the overlap between the O_2_ molecular orbitals and the surface U d/f orbitals, which is the most favorable initial configuration for dissociative chemisorption. For the Brillouin zone k-point [29,30] sampling, for the calculation of the UN unit cell, a 7 × 7 × 7 k-point mesh is employed. For the UN(100), (110) surface and adsorption models, the k-point mesh is set to 7 × 7 × 1. For the (111) surface and adsorption models, the k-point mesh is set to 5 × 5 × 1. All calculations were performed in a spin-polarized manner (ISPIN = 2). A ferromagnetic (FM) ordering of the U 5f moments was assumed, which is a common simplification adopted in previous DFT + U studies of UN surfaces [9,17,26] and has been shown to have a negligible effect on the surface and adsorption energetics of interest here.

The essence of surface energy is the energy required to break intermolecular chemical bonds when a substance creates a new surface. Alternatively, surface energy can be understood as the excess energy possessed by the surface of a material compared to its bulk interior [31]. The surface energy of $E_{\mathrm{surf}\left(UN\right)}$ is defined as:

(1) $$E_{\mathrm{surf}\left(UN\right)}=\frac{1}{2A}\left(E_{\mathrm{slab}}-NE_{\mathrm{bulk}}\right)$$

where $E_{\mathrm{slab}}$ is the total energy of the surface slab model, $E_{\mathrm{bulk}}$ is the energy of a single unit cell, N is the number of unit cells contained in the slab model, and A is the surface area.

Adsorption energy [32,33] refers to the energy absorbed or released when an atom or molecule adsorbs onto a surface, and it is an important indicator of the strength of the interaction between the molecule/atom and the target surface. A negative adsorption energy indicates that the adsorption process releases energy, while a positive adsorption energy indicates that the adsorption process absorbs energy. The adsorption energy is calculated using the following formula:

(2) $$E_{\mathrm{ads}}=E_{\mathrm{slab}+O}-E_{\mathrm{slab}}-E_{O}$$

where $E_{\mathrm{ads}}$ is the adsorption energy, $E_{\mathrm{slab}+O}$ is the total energy of the system after adsorption, $E_{\mathrm{slab}}$ is the energy of the substrate before adsorption, and $E_{O}$ denotes half the energy of a gas-phase O_2_ molecule ($E_{O}$ = ½ E(O_2_)), which is the reference state for a single adsorbed O atom. For O_2_ molecular adsorption, $E_{O}$ is instead replaced by the energy of a gas-phase O_2_ molecule, *E(O*_2_*)*.

Based on the CHGCAR file obtained from self-consistent calculations, charge density difference analysis of the O atom adsorption system on the UN surface can be performed using the VESTA 3.90.5a software, thereby analyzing the charge transfer [32] before and after adsorption. The charge density difference is calculated using the following formula:

(3) $$\Delta \rho =\rho_{\left[O/\mathrm{UN}\right]}-\rho_{O}-\rho_{\mathrm{UN}}$$

where $\rho_{O/\mathrm{UN}}$, $\rho_{O}$, and $\rho_{\mathrm{UN}}$ represent the charge density distributions of the stable O atom adsorption system, the isolated O atom, and the bare UN surface, respectively. Through the calculation and analysis of the charge density difference, the charge transfer during the bonding and bond electron coupling process, as well as the bond polarization direction, can be clearly obtained, which facilitates the understanding of the adsorption process.

Bader charge [34] analysis is performed based on the results of self-consistent calculations to provide further quantitative analysis of charge transfer. The number of transferred electrons can be obtained by subtracting the total number of valence electrons from the pseudopotential information. Bader charge analysis can quantitatively characterize the amount of charge transfer and analyze the charge transfer of atoms in each surface layer.

The work function [35] is defined as the minimum energy required to remove an electron from the solid surface instantaneously. The work function is not an intrinsic property of the bulk material; rather, it is a characteristic of the material surface (e.g., the exposed crystal plane and the degree of contamination). As an important property of metals, the magnitude of the work function is typically about half the ionization energy of a free metal atom. The formula for calculating the surface work function is:

(4) $$\Phi =E_{\mathrm{vacuum}}-E_{\mathrm{Fermi}}$$

where Φ, $E_{\mathrm{vacuum}}$, and $E_{\mathrm{Fermi}}$ represent the work function, vacuum level, and Fermi level, respectively, with units in eV. It should be noted that all calculations in the present study were performed at 0 K; consequently, finite-temperature effects are not considered, and their investigation is reserved for future work, where ab initio molecular dynamics simulations will be employed.

## 3. Result and Discussions

### 3.1. Surface Model and Energy

The UN unit cell adopts the NaCl structure with space group $Fm\bar{3}$*m*. The UN surface models are constructed based on the UN unit cell model, which can be obtained from the Materials Project website. According to the face-centered cubic structure of UN, the unit cell parameters are a = b = c = 0.489 nm and α = β = γ = 90°. After optimization, the lattice constant of UN is calculated to be 4.893 Å, which deviates from the experimental value of 4.886 Å by less than 0.5%, indicating that the selected functional and pseudopotential are reliable.

**Figure 1:**
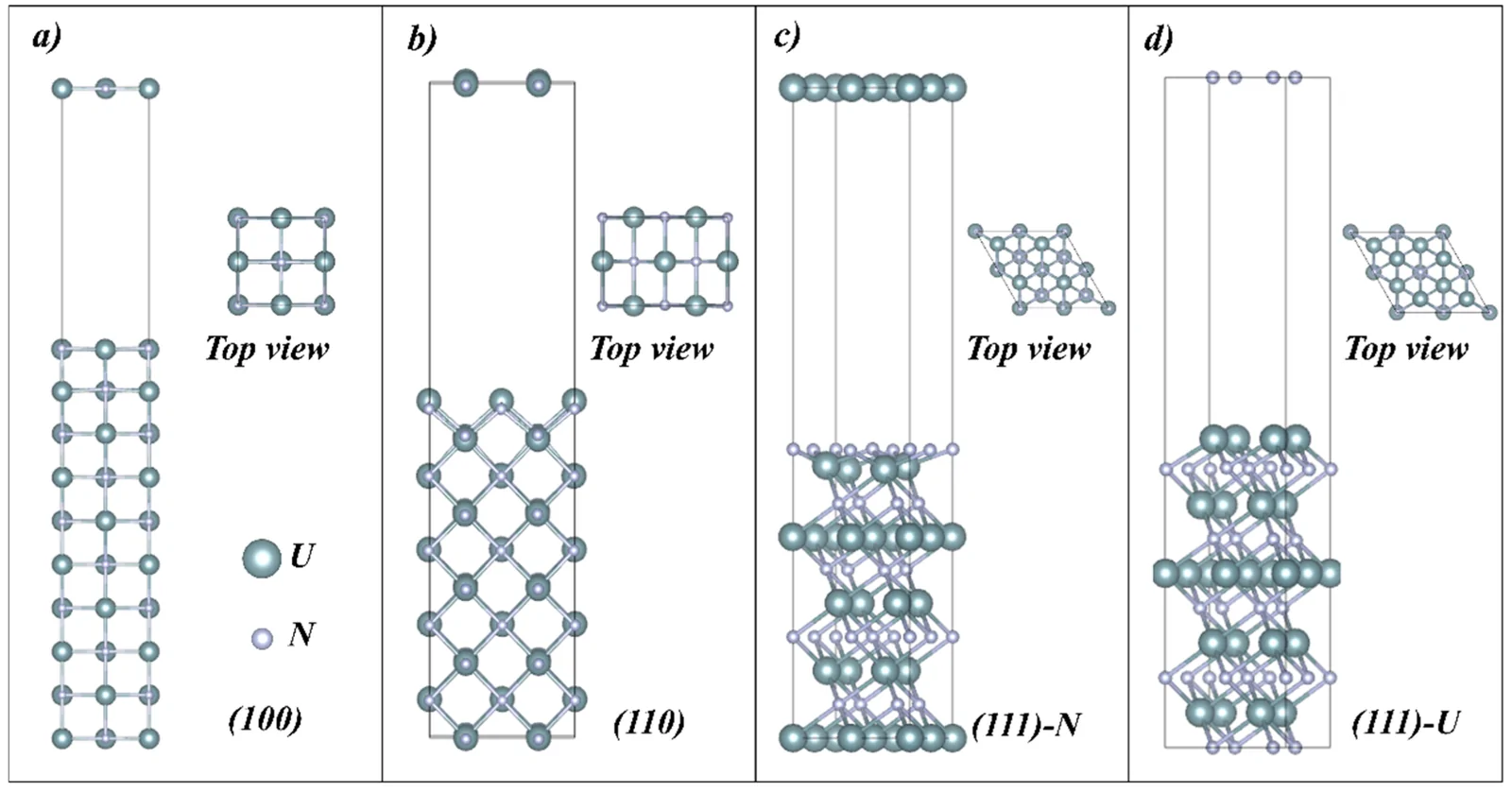
Side and top views of different UN surface slab models: (**a**) (100) surface; (**b**) (110) surface; (**c**) (111)-N surface; (**d**) (111)-U surface.

Figure 1 shows four different UN surfaces: (100), (110), (111)-N, and (111)-U, among which the (111) surface exhibits both a N-terminated and a U-terminated surface. Table 1 presents the calculated surface energies of the four UN surfaces. For the (111) surface, the surface energy of the N-terminated surface is 0.140 eV/Å^2^, which is significantly lower than that of the U-terminated surface (0.283 eV/Å^2^). Previous studies have shown that a lower surface energy facilitates crystal face growth, thereby occupying a larger surface area and making it more suitable as a substrate for adsorption [36]. Therefore, subsequent calculations for the (111) surface are performed on the N-terminated surface. The (111) surface is polar (Tasker type III); the work-function change upon adsorption, which is the quantity of primary interest here, is determined by the local charge transfer and is insensitive to the treatment of the compensating surface dipole. To validate our results, the calculated surface energies are compared with the recent DFT study of Liu et al. [37], who reported surface energies for the stoichiometric UN(001), UN(110), and N-terminated UN(111) surfaces. After unit conversion (1 J/m^2^ = 0.0624 eV/Å^2^), our (100) surface energy (0.091 eV/Å^2^) is in excellent agreement with their UN(001) value of 1.48 J/m^2^ (0.092 eV/Å^2^), confirming the reliability of our slab model and computational parameters. The (110) and (111) surface energies are likewise of the same order of magnitude as those reported by Liu et al., with the remaining quantitative differences attributable to differences in slab thickness, surface termination, and the treatment of the polar (111) surface.

### 3.2. Adsorption of Oxygen Molecule and Atom

**Figure 2:**
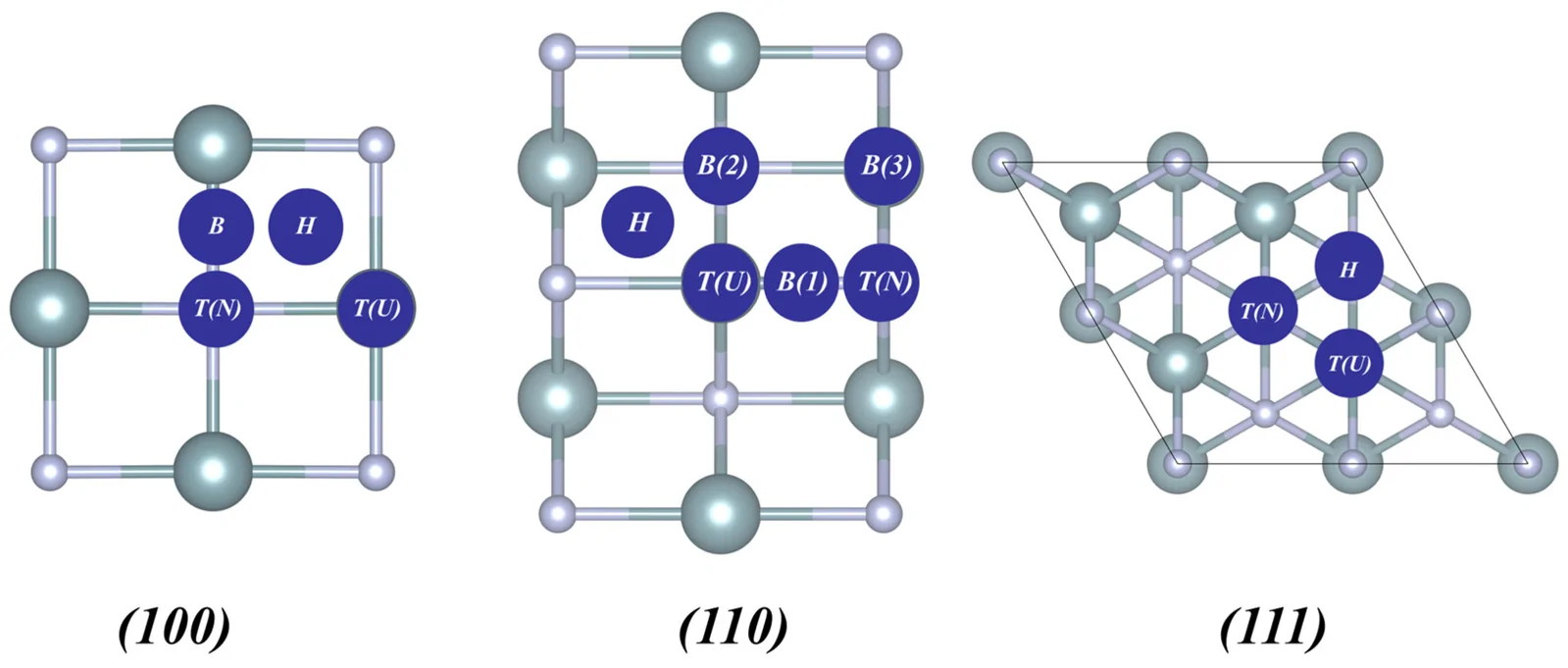
Different adsorption sites on various UN surfaces.

During the initial oxidation process, the adsorption of O_2_ molecules and their dissociation into O atoms on the UN surface are two important steps. In this work, four highly symmetric adsorption sites on the UN(100) surface, six on the (110) surface, and three on the (111) surface were investigated. Among them, T(U) denotes the top site of a U atom, T(N) the top site of an N atom, H the hollow site, and B the bridge site between U and N atoms. The (110) surface features three asymmetric bridge sites B, namely, the bridge site between two U atoms, the bridge site between a U and an N atom, and the bridge site between two N atoms. The different adsorption sites on the three UN surfaces are shown in Figure 2. By placing O_2_ molecules at the aforementioned total of 13 adsorption sites and performing relaxation calculations, the adsorption energies of O_2_ molecules in various adsorption configurations were obtained, thereby determining the stability of O_2_ molecules on different surfaces and under different configurations.

Table 2 presents the adsorption energy $E_{\mathrm{ads}}$ of each configuration on different UN surfaces, the shortest distance $d_{U\text{-}O}$ between the two oxygen atoms and the nearest U atom on the UN surface, as well as the distance between the two oxygen atoms (O–O). The adsorption energies of all configurations are negative, indicating that these processes are exothermic and can proceed spontaneously [38].

**Figure 3:**
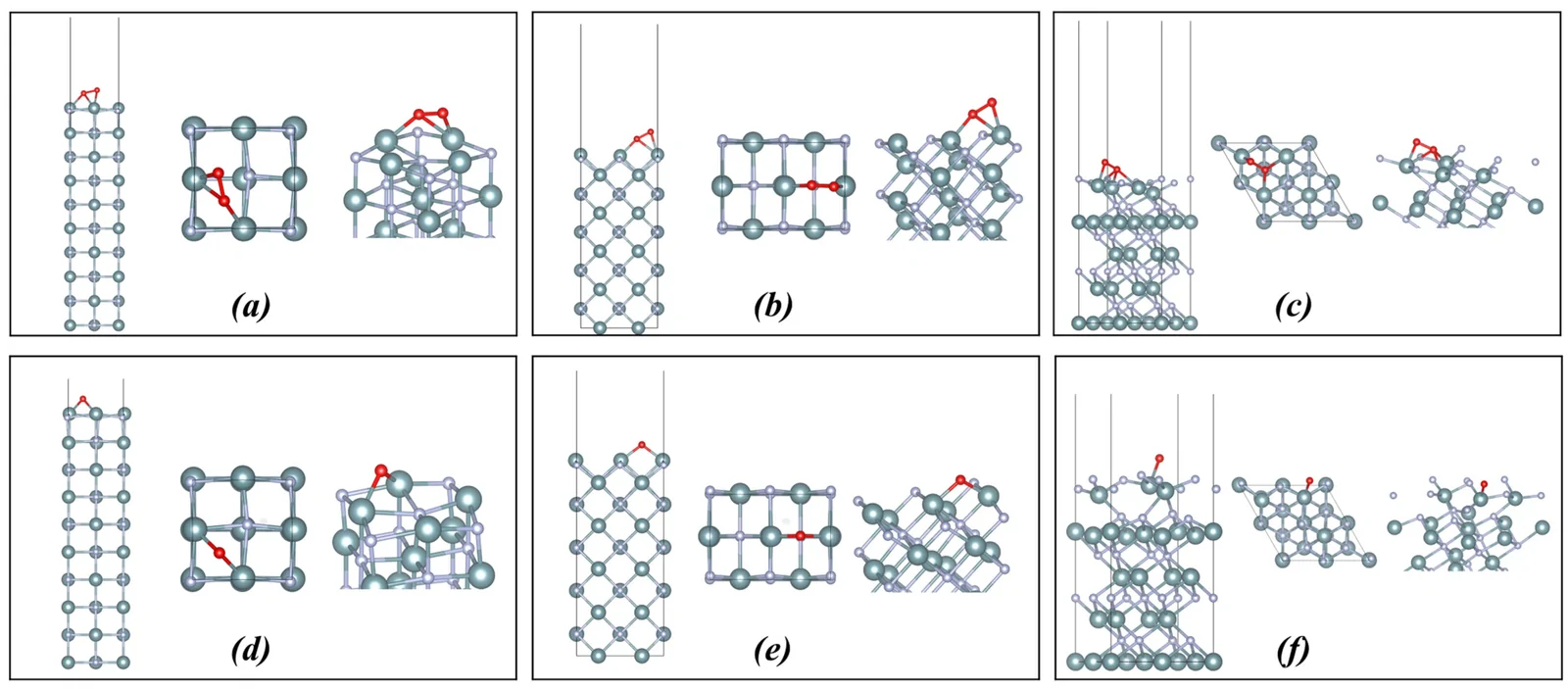
Side views, top views, and enlarged views of the oxygen adsorption sites for the most stable adsorption configurations of O_2_ molecules and O atoms on different UN surfaces: (**a**) (100)-O_2_ hollow site (H); (**b**) (110)-O_2_ bridge site B(2); (**c**) (111)-O_2_ hollow site (H); (**d**) (100)-O hollow site (H); (**e**) (110)-O bridge site B(2); (**f**) (111)-O top site of U atom T(U).

For the (100) surface, the hollow site (H) exhibits the largest absolute adsorption energy (−4.05 eV), making it the most stable adsorption site for O_2_ molecules on the UN(100) surface. In addition, adsorption at the top site of the N atom [T(N)] is unstable; upon optimization, the O_2_ molecule relaxes to the bridge site, with the two O atoms positioned on opposite sides of the bridge. For the (110) surface, the bridge site between two U atoms B(2) has the largest absolute adsorption energy (−4.81 eV) and is the most stable adsorption site for O_2_ molecules. After optimization, all six initial adsorption configurations relax to the same bridge site B(2) between two surface U atoms, regardless of their starting positions. Combined with its largest absolute adsorption energy of −4.81 eV (Table 2), this identifies the B(2) bridge site as the most stable adsorption site among the configurations investigated for O_2_ on the UN(110) surface. Analysis indicates that, due to the groove-like structure of the (110) surface, the two O atoms in the most stable O_2_ adsorption configuration are embedded in the bridge sites within the groove, suggesting that the groove structure of the (110) surface provides a richer coordination environment that facilitates stable O atom adsorption. For the (111) surface, the hollow site (H) has the largest absolute adsorption energy (−5.73 eV) and is the most stable adsorption position. At this site, the U–O bond lengths are 2.094 Å and 2.097 Å, respectively, significantly shorter than those on the (100) and (110) surfaces. Shorter U–O bond lengths generally imply stronger bonding [39], which is highly consistent with the adsorption energy data. After optimization, the U atoms closest to the adsorbed oxygen undergo a noticeable outward (vertical) displacement toward the oxygen atoms. Such an upward relaxation of the surface U atoms is consistent with the early stage of surface corrosion, in which U atoms are progressively drawn out of the surface lattice. Moreover, the hollow site on the (111) surface allows each O atom to coordinate simultaneously with three surface U atoms, resulting in stronger U–O interactions. The most stable adsorption configurations on each UN surface are shown in Figure 3.

For the O_2_ adsorption models on the three surfaces, the O_2_ molecules have already dissociated after optimization, with the O–O bond length is significantly stretched from 1.23 Å in gaseous O_2_ to approximately 1.44–1.47 Å, far exceeding the criterion for the superoxide state (O_2_^−^, ~1.33 Å) and approaching that of a peroxide anion (O_2_^2−^, ~1.49 Å) [40], approaching complete dissociation. This elongated O_2_ species, which is intermediate between a superoxide and full dissociation, is therefore referred to as a peroxo-like state. This is highly consistent with the conclusions of Bo et al. [9], indicating that UN surfaces exhibit high reactivity toward O_2_. The order of O_2_ adsorption strength on UN surfaces is: UN(111) H site (−5.03 eV) > UN(110) B(2) site (−4.81 eV) > UN(100) H site (−4.05 eV). This can be attributed to differences in surface structure and coordination environment. The three-coordinated hollow site on the (111) surface provides the strongest binding, followed by the groove bridge site on the (110) surface, while the hollow site on the flat (100) surface is relatively the weakest. This phenomenon is in good agreement with the conclusion of Su et al. [41] regarding the preferential adsorption at hollow sites on the α-U(001) surface, as well as the finding of Li et al. [39] that O atoms preferentially occupy hollow sites. All these findings reflect the stabilization tendency of O atoms to maximize their coordination number with U atoms.

Table 3 lists the adsorption energies and equilibrium heights of O atoms in 13 configurations on the three UN surfaces. Compared with O_2_ molecular adsorption, the absolute values of O atom adsorption energies are significantly larger. This difference arises because O_2_ molecules must first break the O–O bond before forming U–O bonds, a process that consumes energy [40] spontaneous dissociative chemisorption at 0 k. In contrast, O atoms bond directly with the surface without needing to overcome a dissociation barrier.

For O atom adsorption, on the (100) surface, the hollow site (H) exhibits the largest absolute adsorption energy (−6.97 eV), indicating that it is the most stable adsorption configuration on the (100) surface. On the (110) surface, the U–U bridge site B(2) has the largest negative adsorption energy (−8.22 eV), and all six adsorption configurations relax to this site after optimization, which is consistent with the results of O_2_ adsorption on the (110) surface, indicating that the most stable adsorption site for O atoms is the U–U bridge site. The adsorption energies of all six configurations fall within the range of −7.1 to −8.2 eV, substantially more negative overall than those on the (100) surface (−5.2 to −7.0 eV), with the energy ranges shifted by approximately 2 eV toward stronger binding. This contrasts with the ~1 eV difference in the most stable O_2_ adsorption energies between the two surfaces, indicating that the groove structure of the (110) surface is even more effective in capturing O atoms than O_2_ molecules. On the (111) surface, the configuration initially placed at the T(U) top site yields the largest negative adsorption energy (−5.09 eV). However, after optimization the O atom relaxes away from the ideal top position toward the neighboring surface N atoms, rather than remaining at T(U). This migration indicates that the T(U) top site is not a deep local minimum, further confirming that O atom adsorption on this surface is relatively weak compared with the (110) and (100) surfaces.

The order of O atom adsorption strength on UN surfaces is: UN(110) B(2) site (−8.22 eV) > UN(100) H site (−6.97 eV) > UN(111) T(U) site (−5.09 eV), which differs from the order observed for O_2_ molecular adsorption. This discrepancy indicates that the adsorption of O_2_ molecules and O atoms is governed by different factors. First, O_2_ molecular adsorption requires both O atoms to coordinate simultaneously with the surface; thus, the three-coordinated hollow site on the (111) surface can accommodate both O atoms simultaneously, allowing each to bond with three U atoms, giving O_2_ adsorption on the (111) surface the greatest advantage. Second, for isolated O atom adsorption, the advantage of the groove structure on the (110) surface becomes prominent—the O atom can embed itself into the groove, forming multiple U–O bonds with U atoms on both sides of the groove. Meanwhile, the geometric confinement effect of the groove “locks” the O atom near the surface in the vertical direction, enhancing bond strength and giving O atom adsorption on the (110) surface a significant advantage. Third, for a single O atom, the three-coordinated hollow site on the (111) surface provides excessive coordination, and after optimization the O atom moves away from the neighboring N atoms toward the surface U atoms, resulting in relatively weak adsorption. Consequently, the (111) surface exhibits the weakest O atom adsorption capacity.

### 3.3. Electronic Structure of the Adsorption System

To further investigate the adsorption mechanisms of O_2_ molecules and O atoms on different UN surfaces, the electronic structures of the adsorption systems need to be studied, including charge density difference analysis, Bader charge analysis, density of states (DOS) analysis, and surface work function analysis.

**Figure 4:**
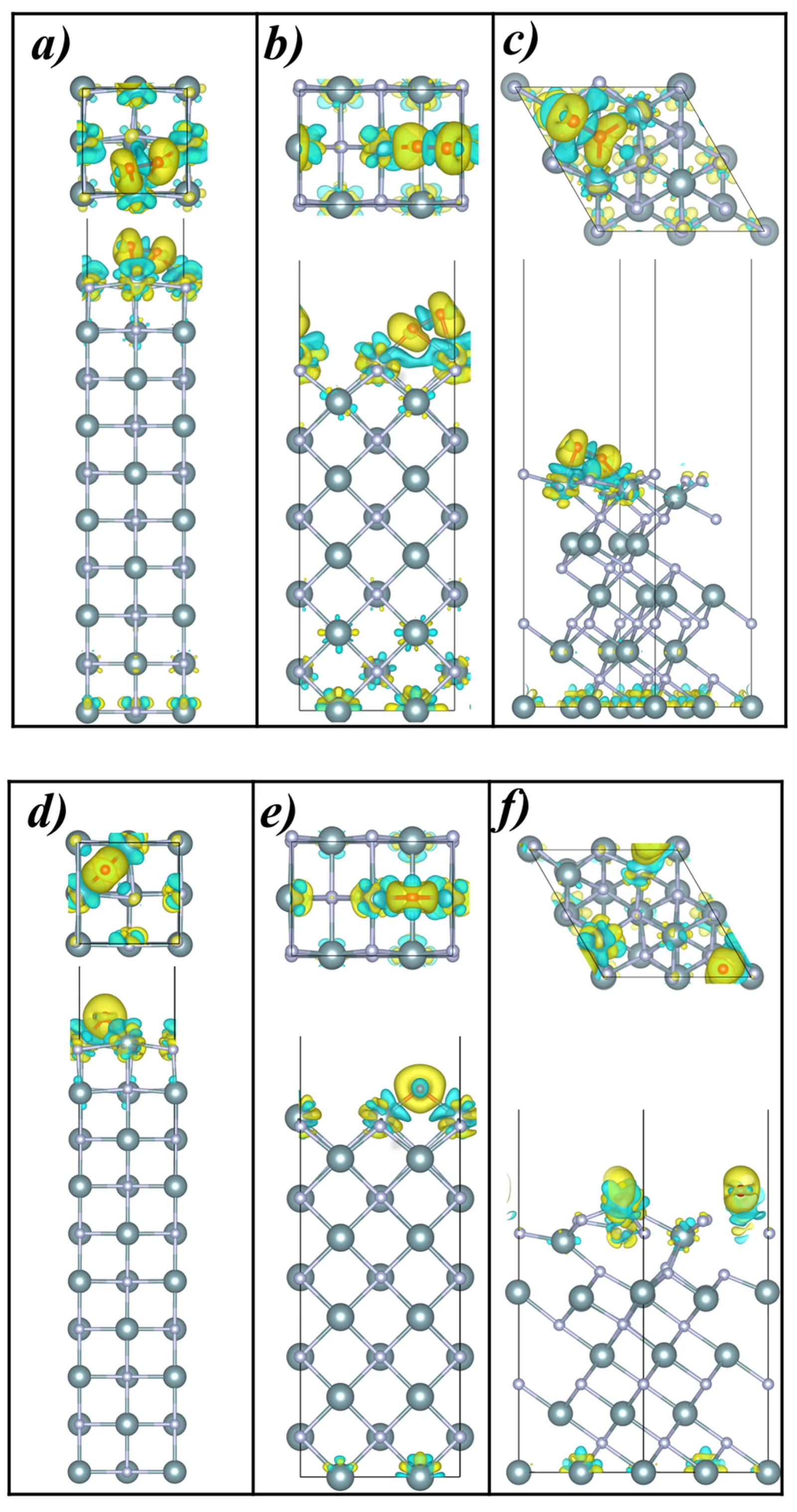
Side views and top views of charge density difference for the most stable adsorption configurations of O_2_ molecules and O atoms on different UN surfaces: (**a**) (100)-O_2_ hollow site (H); (**b**) (110)-O_2_ bridge site B(2); (**c**) (111)-O_2_ hollow site (H); (**d**) (100)-O hollow site (H); (**e**) (110)-O bridge site B(2); (**f**) (111)-O top site of U atom T(U).

The charge density difference diagrams of the most stable adsorption configurations of O_2_ molecules and O atoms on UN(100), (110), and (111) surfaces are shown in Figure 4. In the figures, the yellow regions represent electron accumulation, while the blue regions represent electron depletion. The charge density difference analysis reveals the electron gain or loss of each atom.

In all six figures, the O atoms are surrounded by yellow regions, the surface N atoms are mostly covered by yellow regions, while the surface U atoms, though covered by both yellow and blue regions, are predominantly surrounded by blue regions. This indicates that the charge density of O and N atoms increases, while that of U atoms decreases, with electrons transferring from the surface U atoms to the O atoms and surface N atoms. It can be seen from the figures that charge transfer occurs almost exclusively between the O atoms and the topmost surface atoms of UN. For the O_2_ molecular adsorption systems, a blue cloud appears at the O–O bond, indicating that the chemical bond between the O atoms loses electrons and undergoes dissociation. For all six adsorption configurations, the two U atoms closest to the O atom on the UN surface exhibit significant charge depletion, indicating that the O atom tends to bond preferentially with two surface U atoms. This is consistent with the aforementioned finding that almost all O_2_ molecules and O atoms are stabilized at bridge or hollow sites after optimization, further confirming that O preferentially coordinates simultaneously with two or more U atoms.

The charge distribution and transfer data of atoms in each layer require further analysis of the model. Since O_2_ dissociates on the UN surface and subsequently undergoes further adsorption and diffusion as individual O atoms, a qualitative investigation of the charge distribution for O atom adsorption is needed in the subsequent study. Bader charge analysis, performed on the basis of self-consistent calculation results, provides further quantitative analysis of charge transfer. The calculated electron distribution and transfer results for O atom adsorption at the UN(100) H site, UN(110) B(2) site, and UN(111) T(U) site are presented in Table 4.

In Table 4, $q_{1\mathrm{st}}$, $q_{2\mathrm{nd}}$, $q_{3\mathrm{rd}}$, $q_{4\mathrm{th}}$, and $q_{5\mathrm{th}}$ represent the sum of transferred electrons for four U atoms and four N atoms in each of the first to fifth layers of the UN surface, respectively, where $q_{5\mathrm{th}}$ corresponds to the bottommost layer and $q_{1\mathrm{st}}$ to the topmost layer. $q_{U}$, $q_{N}$, and $q_{O}$ denote the sum of transferred electrons for all U atoms, N atoms, and oxygen atoms in the model, respectively. For the (100)-O adsorption system, the Bader charge analysis shows that the calculated Bader charges of the first to fifth atomic layers in the UN(100) surface model are −0.07, 0.02, −0.01, 0.14, and −1.20, respectively, while those of the U and N atoms are −33.47 and 33.47, respectively. After O atom adsorption, the Bader charges of the U atoms, N atoms, and O atom are −33.96, 32.84, and 1.11, respectively. Comparing the data of the surface model and the adsorption model, it is found that after O atom adsorption on the UN surface, the O atom gains 1.12 e, indicating that the UN surface transfers 1.12 e to the O atom during the adsorption process. Examining the Bader charges of each layer in the adsorption model, the first-layer atoms exhibit the largest charge transfer of −1.20 e, while the remaining layers lose fewer electrons, suggesting that charge transfer mainly occurs in the first atomic layer. Similarly, for the (110)-O adsorption system, the calculations show that, compared with the UN(110) surface model, the O atom gains 1.13 e after adsorption, with the UN surface transferring 1.13 e to the O atom. The first-layer atoms again exhibit the largest charge transfer (−1.31 e), whereas the remaining layers lose fewer electrons, indicating that charge transfer also predominantly occurs in the first layer. For the (111)-O adsorption system, the calculations show that, compared with the UN(111) surface model, the O atom gains 1.07 e after adsorption, and the N atoms transfer 1.12 e to the O atom while also transferring electrons to the U atoms. Analyzing the charge transfer of each layer, it is found that the first, second, and fifth atomic layers exhibit relatively large charge transfers of −2.96 e, −1.52 e, and 3.40 e, respectively. It is inferred that this adsorption model undergoes a relatively large overall charge transfer, with charge transfer being particularly pronounced.

**Figure 5:**
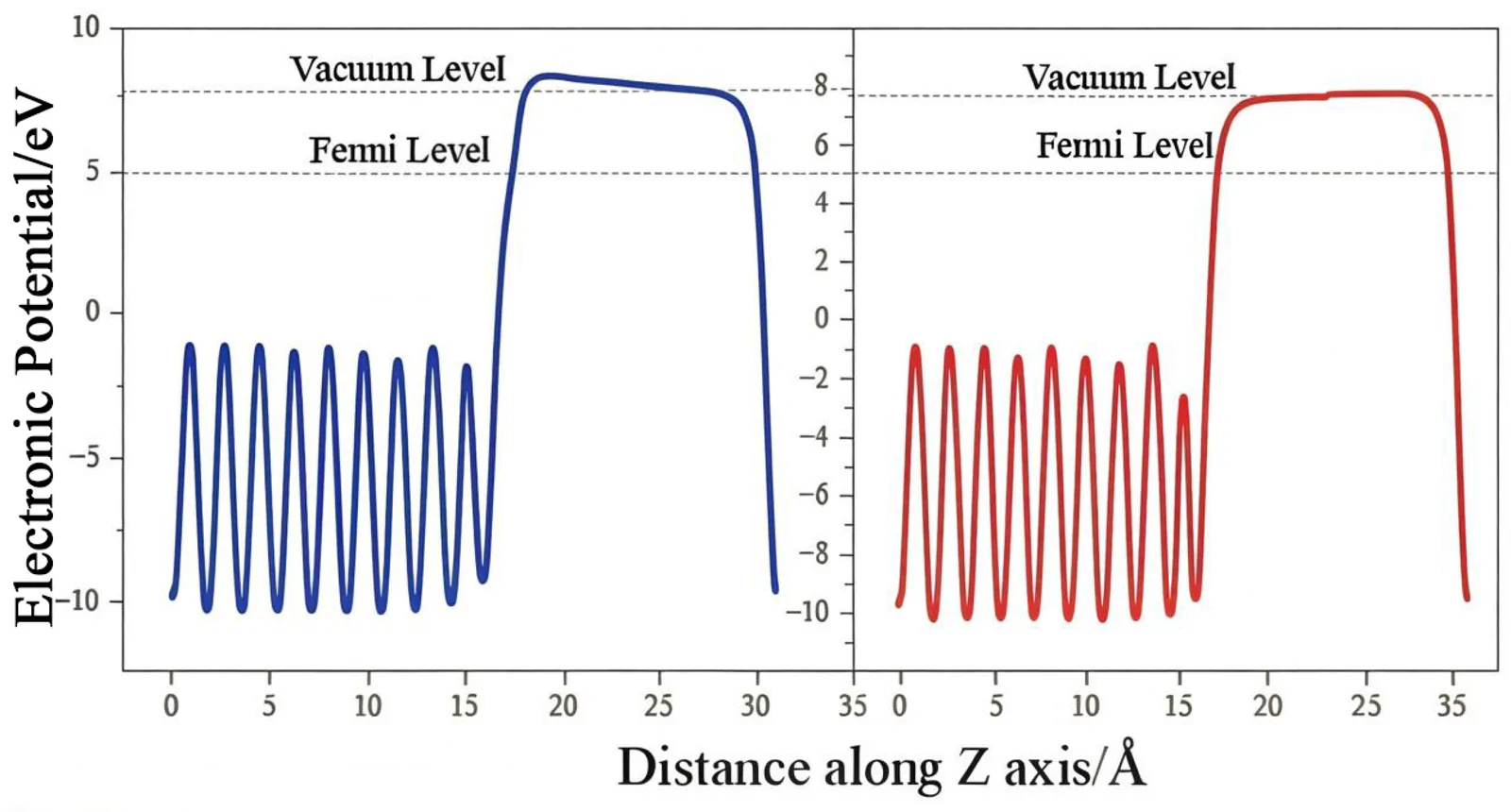
Variation in the electrostatic potential along the Z-axis for the UN(100)-O adsorption system. The red line represents the vacuum electrostatic potential of the clean UN surface, and the blue line represents the vacuum electrostatic potential of the surface after O atom adsorption.

**Figure 6:**
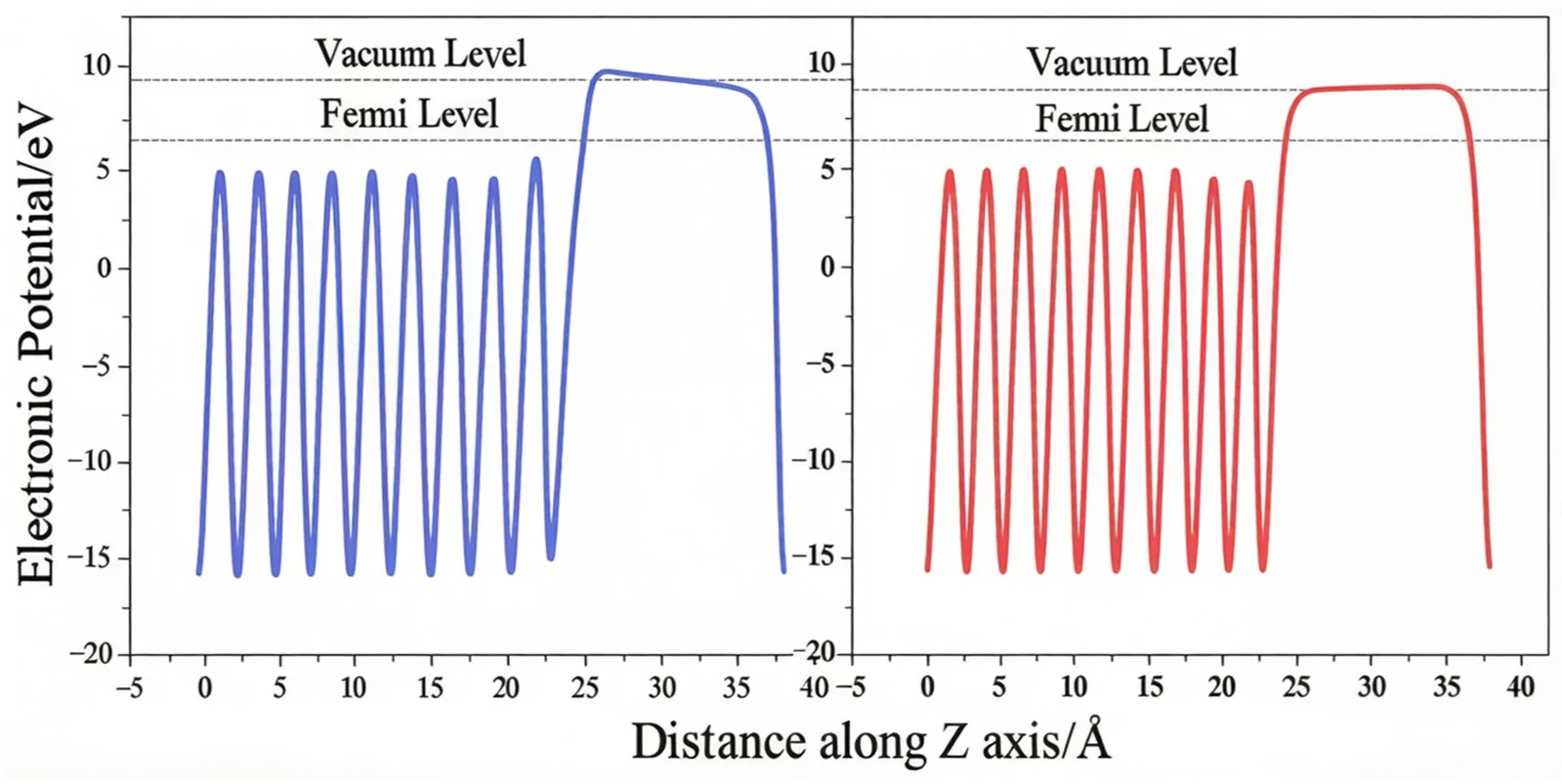
Variation in the electrostatic potential along the Z-axis for the UN(110)-O adsorption system. The red line represents the vacuum electrostatic potential of the clean UN surface, and the blue line represents the vacuum electrostatic potential of the surface after O atom adsorption.

**Figure 7:**
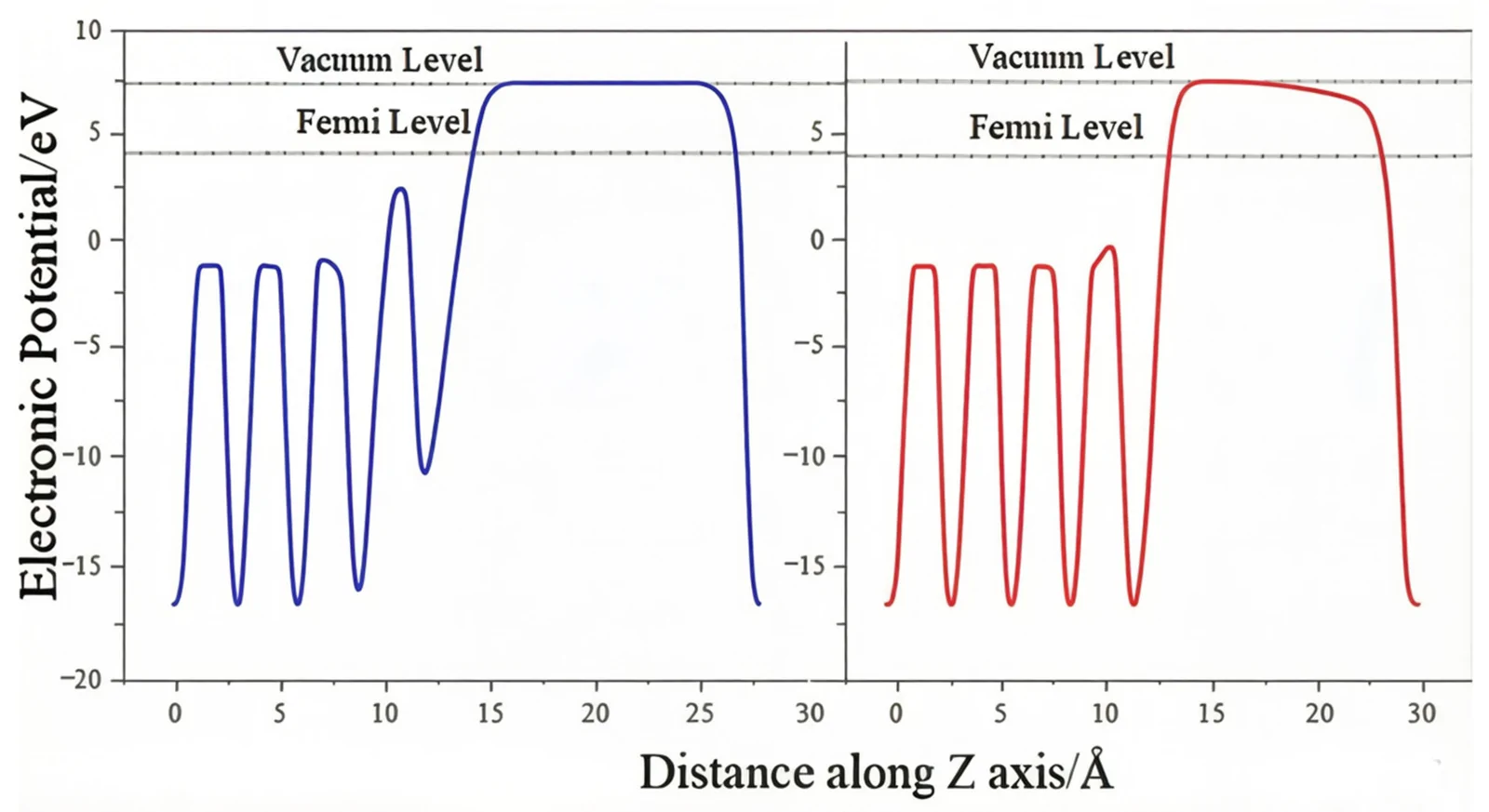
Variation in the electrostatic potential along the Z-axis for the UN(111)-O adsorption system. The red line represents the vacuum electrostatic potential of the clean UN surface, and the blue line represents the vacuum electrostatic potential of the surface after O atom adsorption.

By processing the vacuum electrostatic potential and Fermi level data obtained from self-consistent calculations, the variation curves of the electrostatic potential along the Z-axis before and after O atom adsorption on the UN surface can be obtained, as shown in Figure 5, Figure 6 and Figure 7. The right panel shows the vacuum electrostatic potential of the clean UN surface, and the left panel shows that of the surface after O atom adsorption. Combined with the formula for calculating the surface work function, the changes in the surface work function before and after O atom adsorption are obtained, as presented in Table 5.

By comparing the data, it can be observed that after O atom adsorption on the UN surface, the work functions of the (100) and (110) surface adsorption models increase to some extent, rising by 0.62 eV and 0.39 eV, respectively, relative to their clean surface models. This indicates that the surface after O atom adsorption is less prone to losing electrons compared with the clean surface. For the O atom adsorption model on the UN(111) surface, the work function decreases by 0.57 eV after O atom adsorption relative to the clean surface model, suggesting that this surface becomes more prone to losing electrons after O atom adsorption, and the O atom is more likely to undergo further reactions with the surface in this adsorption configuration.

Combining the Bader charge analysis and work function analysis, the following conclusions can be drawn: (1) In all three UN surfaces, the O atom acts as the electron acceptor during adsorption, with charge transfer amounts in the range of 1.07–1.13 e, which is significantly larger than that of O_2_ molecular adsorption (approximately 0.1–0.2 e), indicating that O atom adsorption is a strong chemisorption rather than physisorption. (2) The charge transfer amount is positively correlated with the adsorption energy: (110) > (100) > (111), suggesting that charge transfer is an important factor driving the adsorption energy. (3) The work function analysis shows that the work function increases after O atom adsorption on the (100) and (110) surfaces, while it decreases on the (111) surface, with the latter being more favorable for further reactions of the O atom.

The density of states (DOS) [42] can be used to quantitatively study the hybridization characteristics of electronic orbitals between the adsorbate and surface atoms, revealing the adsorption mechanism and bonding nature at the electronic structure level, in order to further investigate the interactions between U atoms and between U and O atoms. Based on the above analysis, O atoms gain electrons from U atoms and form bonds with U atoms. Therefore, this work selects the six adsorption configurations identified above, and performs DOS analysis on the O_2_ molecule/O atom and their nearest U atoms, with particular focus on the hybridization characteristics between the O-2p orbital and the U-5f and N-2p orbitals.

**Figure 8:**
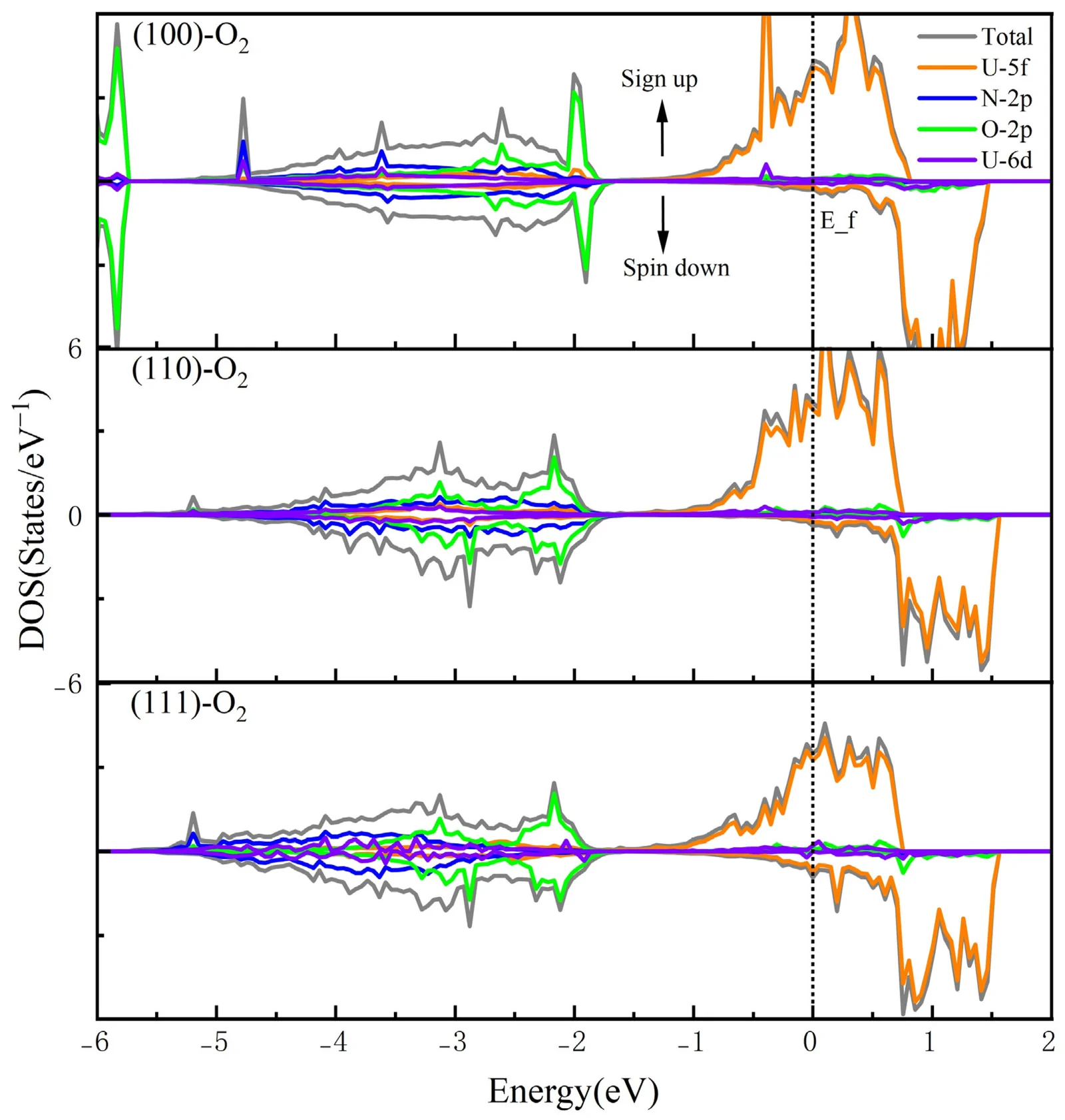
Partial density of states (PDOS) of O_2_ molecule adsorption on different UN surfaces.

Figure 8 shows the PDOS of the O-2p orbital of the O_2_ molecule together with the nearest-neighbor U-5f and U-6d orbitals at the adsorption site after O_2_ adsorption at the bridge site on UN(100), the bridge site on UN(110), and the hollow site on UN(111). The total density of states (TDOS) exhibits a continuous distribution near the Fermi level, confirming that the UN surface retains metallic conductive character after O_2_ adsorption. Significant differences in spin polarization intensity are observed among the three surfaces: the UN(100)-O_2_ system has a spin polarization of 4.03 states/eV at the Fermi level (spin-up = 4.32, spin-down = 0.29); the net spin DOS of UN(110)-O_2_ is 3.61 states/eV (spin-up = 4.01, spin-down = 0.40); and the net spin DOS of UN(111)-O_2_ is the smallest at 2.55 states/eV (spin-up = 3.49, spin-down = 0.94).This sequence indicates that the spin polarization effect is most pronounced on the UN(100) surface. For the O-2p orbital, the O-2p DOS on all three surfaces is highly localized in the valence band region of −6 to −4 eV, exhibiting sharp resonance peak structures. The contribution of the O-2p DOS near the Fermi level is almost negligible, indicating that all O-2p electrons are involved in bonding with surface U atoms and do not participate in the conduction process. In contrast, the U-5f orbital has its main electronic states concentrated in a narrow region of −0.5 to +0.5 eV near the Fermi level, forming an extremely sharp peak structure. The main U-5f peak of UN(100)-O_2_ is located at −0.4 eV with a peak value of 8.32 states/eV; the main U-5f peak of UN(110)-O_2_ is at −0.15 eV with a peak value of 4.41 states/eV, but there is also a peak at +0.10 eV with a high intensity of 7.84 states/eV; the main U-5f peak of UN(111)-O_2_ is at −0.05 eV with a peak value of 3.82 states/eV. Notably, in the energy range where the O-2p main peak is located, the contribution of U-5f is extremely weak, indicating that direct hybridization between O-2p and U-5f in O_2_ molecular adsorption is very limited. Therefore, this work suggests that the primary bonding channel should be direct p–d hybridization between O-2p and U-6d, while U-5f participates indirectly in the bonding process through f–d orbital coupling with U-6d.

**Figure 9:**
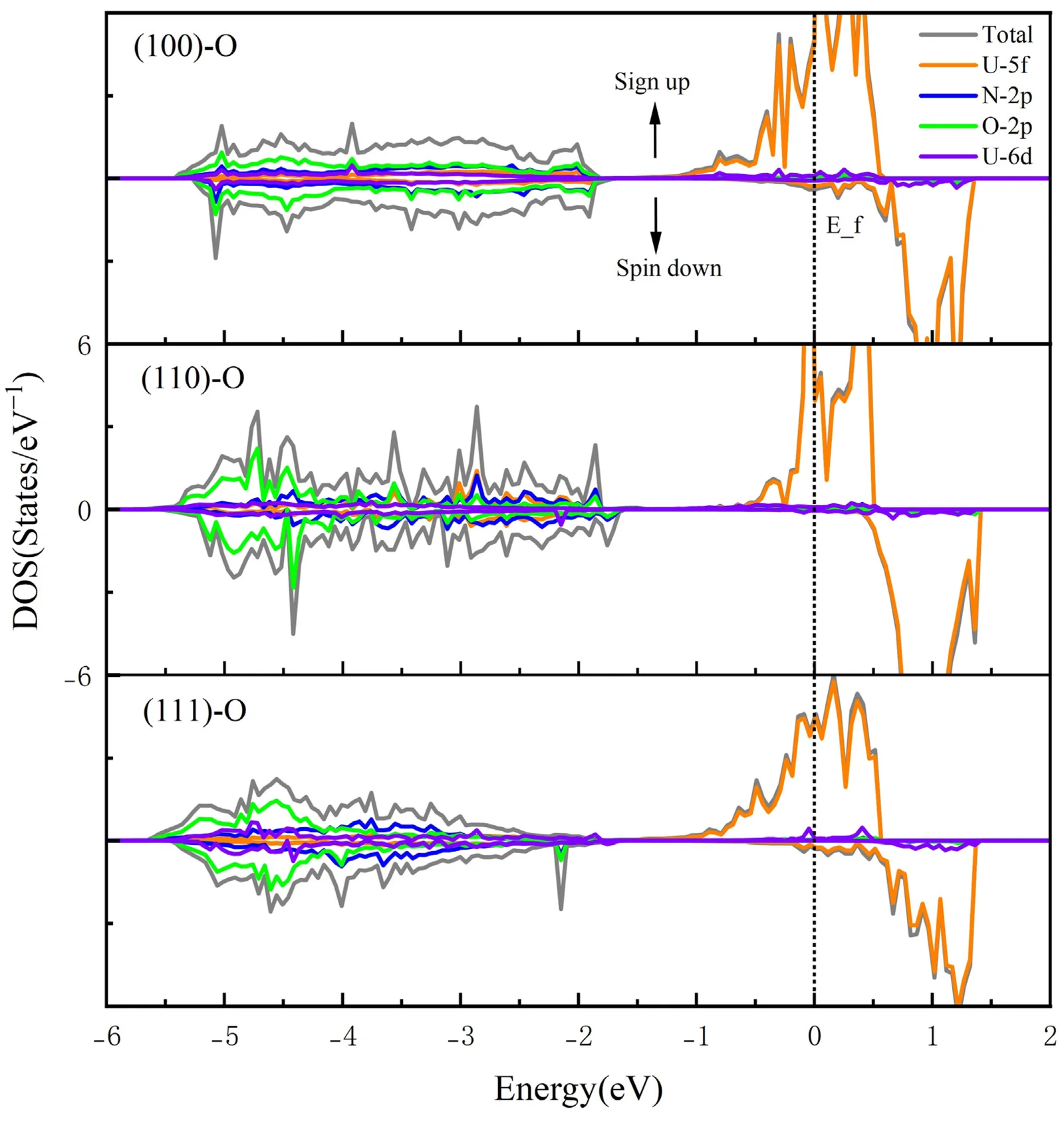
Partial density of states (PDOS) of O atom adsorption on different UN surfaces.

Figure 9 shows the PDOS spectra of a single O atom adsorbed at its most stable site on each surface, namely the hollow site (H) on UN(100), the bridge site B(2) on UN(110), and the U top site T(U) on UN(111)—together with the nearest-neighbor U-5f and U-6d orbitals. Compared with O_2_ molecular adsorption, the electronic structure of the O atom adsorption system undergoes fundamental changes. Taking UN(100)-O as an example, the total DOS at the Fermi level has a spin-up DOS of 5.02 states/eV and a spin-down DOS of 0.41 states/eV, with the net spin DOS increasing to 4.61 states/eV, higher than the value of 4.03 states/eV for molecular adsorption. The characteristics of the O-2p orbital differ significantly from those in the O_2_ molecular adsorption system. Under O atom adsorption, the distribution range of the O-2p states broadens from −8 to −5 eV for the molecule to −7 to −3 eV, and the main peak intensity is substantially reduced. The O-2p main peak of UN(100)-O is located at −5.03 eV, with spin-up and spin-down peak values of 0.94 states/eV and −1.30 states/eV, respectively—only about one-sixth of that in the molecular adsorption system. This change arises because, in the molecular state, the O–O covalent bond confines the O-2p electrons highly within the molecular orbitals. When the O atom dissociates and adsorbs on the surface, the O-2p orbitals hybridize fully with the U orbitals, forming U–O bonding states distributed over a wider energy range, resulting in lower peak values and broader peak shapes. This confirms that O atoms undergo chemisorption on all three surfaces. The changes in the U-5f orbital are even more dramatic. The U-5f DOS of UN(100)-O exhibits a double-peak structure near the Fermi level: the peak at −0.30 eV reaches 4.83 states/eV, while the peak at +0.05 eV reaches as high as 22.31 states/eV, approximately five times the U-5f peak value of the O_2_ molecular system (4.32 states/eV). This dramatic enhancement of the U-5f electronic DOS indicates that the localization of U-5f electrons is significantly increased after O atom chemisorption, and the formation of U–O bonds substantially alters the local electronic environment of the U atoms. In the O-2p main peak energy range (−5.5 to −4.5 eV), the U-5f contribution is approximately 0.02–0.15 states/eV, which, although still far lower than the O-2p peak intensity, is notably enhanced compared with the molecular system, indicating that the direct p–f hybridization channel between O-2p and U-5f is strengthened after O atom dissociation.

## 4. Conclusions

In this work, the oxidation mechanisms of oxygen on UN(100), (110), and (111) surfaces were investigated through DFT calculations. The surface energies of different surfaces were computed, and the adsorption configurations of O_2_ molecules and O atoms on each surface were constructed, identifying the most stable adsorption configurations of oxygen on different UN surfaces. Subsequently, the electronic structures of the most stable adsorption configurations were calculated, and the bonding between oxygen and the UN surfaces was analyzed.

(1) The UN(100) surface has the lowest surface energy (0.091 eV/Å^2^), followed by (110) (0.127 eV/Å^2^), while the (111)-N-terminated surface (0.170 eV/Å^2^) is the highest and most reactive. The most stable adsorption site for O_2_ is the hollow site (H) on the (100) surface, the bridge site B(2) on the (110) surface, and the hollow site (H) on the (111) surface. The order of O_2_ adsorption strength on UN surfaces is: UN(111) H site > UN(110) B(2) site > UN(100) H site, with the three-coordinated hollow site on the (111) surface providing the strongest binding for oxygen molecules. The order of O atom adsorption energies is: UN(110) B(2) site > UN(100) H site > UN(111) T(U) site, with the (110) surface exhibiting the strongest oxygen trapping capability due to its groove structure. O_2_ dissociation is thermodynamically highly favorable on all three surfaces, with the O–O bond length elongating from 1.23 Å to 1.45–1.47 Å, reaching the peroxo (O_2_^2−^) level. After dissociation, the O atoms exist in the form of strong chemisorption: on the (100) surface, the dissociated O atoms occupy two separate bridge sites; on the (110) surface, they are embedded in the groove bridge sites; and on the (111) surface, they occupy the hollow site with threefold U coordination.
(2) Bader charge analysis shows that O atoms gain 1.07–1.13 e from the UN surface, with the charge transfer following the order: (110) (1.13 e) > (100) (1.11 e) > (111) (1.07 e), which is positively correlated with the adsorption energy. The extent of charge redistribution varies with the crystal face: it is confined to the first layer on the (100) and (110) surfaces, while extending to the first through fifth layers on the (111) surface, reflecting the need for multi-layer synergy on close-packed surfaces.
(3) The work function changes exhibit opposite trends: the (100) and (110) surfaces increase by 0.618 eV and 0.395 eV, respectively, while the (111) surface decreases by 0.569 eV. O atom adsorption on the (111) surface is more reactive and more favorable for subsequent reactions.
(4) O_2_ molecules are initially adsorbed on the UN surface in a peroxo-like state, followed by energy-driven dissociation into O atoms and the formation of U–O covalent bonds. The essence of the electronic structure evolution is the transformation of O-2p orbitals from intramolecular bonding to surface bonding. The primary bonding channel for both systems is p–d hybridization between O-2p and U-6d orbitals, while U-5f participates indirectly through orbital coupling with U-6d. In O atom adsorption, direct p–f hybridization is enhanced but still does not become the dominant bonding mechanism.

## Figures and Tables

**Table 1:** Surface energies of different UN surfaces.

Surface slab models	100	110	111-N	111-U
Surface energy (eV/Å^2^)	0.091	0.127	0.170	0.283

**Table 2:** Adsorption energies of O_2_ molecular adsorption configurations.

Surface	Adsorption Site	*E_ads_* (eV)	*d_u-o_* (Å)	O-O Distance (Å)
(100)	T(N)	−2.29	2.930/3.008	1.275
	B	−3.85	2.324/2.276	1.492
	T(U)	−3.49	2.288/2.309	1.495
	H	−4.05	2.245/2.420	1.469
(110)	T(N)	−4.66	2.281/2.236	1.484
	B(1)	−4.55	2.180/2.357	1.495
	B(2)	−4.81	2.209/2.408	1.448
	B(3)	−4.23	2.209/2.407	1.445
	T(U)	−4.55	2.210/2.411	1.447
	H	−3.49	2.178/2.358	1.500
(111)	T(N)	−2.05	2.737/2.983	1.449
	H	−5.03	2.094/2.097	1.459
	T(U)	−4.93	2.127/2.519	1.458

**Table 3:** Adsorption energies of O atom adsorption configurations.

Surface	Adsorption Site	*E_ads_*(*eV*)	*d_u-surface_*(*Å*)
(100)	T(N)	−6.69	1.368
	B	−5.23	1.677
	T(U)	−5.36	1.880
	H	−6.97	1.371
(110)	T(N)	−8.20	1.281
	B(1)	−7.12	1.292
	B(2)	−8.22	1.283
	B(3)	−7.47	1.299
	T(U)	−7.81	1.279
	H	−7.79	1.285
(111)	T(N)	−4.51	1.579
	H	−5.07	1.862
	T(U)	−5.09	1.879

**Table 4:** Charge distribution and transfer data of the O atom adsorption system.

Adsorption Site	Atoms			*q_total_*(e)	Layers				
	*q_O_*(e)	*q_U_*(e)	*q_N_*(e)		*q5th*(e)	*q4th*(e)	*q3rd*(e)	*q2nd*(e)	*q1st*(e)
(100)surface	-	−33.47	33.47	0	−0.06	0.03	−0.01	0.22	−0.18
(100)-O	1.12	−33.96	32.84	−3.9 × 10^−5^	−0.07	0.02	−0.01	0.14	−1.20
(110)surfcace	-	−33.49	33.49	0	0.10	−0.02	−0.13	0.33	−0.28
(110)-O	1.13	−34.18	33.05	3.49 × 10^−5^	0.10	−0.04	−0.08	0.22	−1.31
(111)surfcace	-	−32.61	32.61	0	3.39	−0.01	0.07	−0.48	−2.97
(111)-O	1.07	−33.30	32.23	3.29 × 10^−5^	3.40	0.05	−0.03	−1.52	−2.96

**Table 5:** Changes in surface work function before and after O atom adsorption.

Model Type	$E_{\mathrm{vacuum}}$/eV	$E_{\mathrm{Fermi}}$	Function/eV	ΔΦ/eV
(100)surface	8.93	6.45	2.48	-
(100)-O	9.41	6.31	3.10	0.62
(110)surfcace	7.73	5.13	2.60	-
(110)-O	7.96	4.96	2.99	0.39
(111)surfcace	7.56	3.93	3.93	-
(111)-O	7.47	4.11	3.36	−0.57

## Data Availability

The original contributions presented in this study are included in the article. Further inquiries can be directed to the corresponding author.
